# Effect of Milk Fermented with *Lactobacillus fermentum* on the Inflammatory Response in Mice

**DOI:** 10.3390/nu10081039

**Published:** 2018-08-08

**Authors:** Lourdes Santiago-López, Adrián Hernández-Mendoza, Verónica Mata-Haro, Belinda Vallejo-Córdoba, Abraham Wall-Medrano, Humberto Astiazarán-García, María del Carmen Estrada-Montoya, Aarón F. González-Córdova

**Affiliations:** 1 Laboratorio de Química y Biotecnología de Productos Lácteos, Centro de Investigación en Alimentación y Desarrollo A. C. (CIAD), Carretera a La Victoria Km. 0.6, Hermosillo, Sonora 83304, Mexico; lulu140288@gmail.com (L.S.-L.); ahernandez@ciad.mx (A.H.-M.); vallejo@ciad.mx (B.V.-C.); carmenes@ciad.mx (M.d.C.E.-M.); 2 Laboratorio de Microbiología e Inmunología, Centro de Investigación en Alimentación y Desarrollo A. C. (CIAD), Carretera a La Victoria Km. 0.6, Hermosillo, Sonora 83304, Mexico; vmata@ciad.mx; 3 Departamento de Ciencias Químico-Biológicas, Instituto de Ciencias Biomédicas, Universidad Autónoma de Ciudad Juárez, Anillo Envolvente del PRONAF y Estocolmo s/n, Ciudad Juárez 32310, Chihuahua, Mexico; awall@uacj.mx; 4 Laboratorio de Patología Experimental, Centro de Investigación en Alimentación y Desarrollo A. C. (CIAD), Carretera a la Victoria Km. 0.6, Hermosillo, Sonora 83304, Mexico; hastiazaran@ciad.mx

**Keywords:** fermented milk, Th1/Th17 response, inflammatory process

## Abstract

Currently, the effect of fermented milk on the T-helper 17 response in inflammatory bowel diseases (IBDs) is unknown. The aim of the present study was to evaluate the effect of milks fermented with *Lactobacillus fermentum* on the Th1/Th17 response in a murine model of mild IBD. Exopolysaccharide (EPS), lactic acid (LA), and total protein (TP) contents and bacterial concentration were determined. Male C57Bl/6 mice intragastrically received either raw (FM) or pasteurized (PFM) fermented milk before and during a dextran sulfate infusion protocol. Blood, spleen, and colon samples were collected at Weeks 6 and 10. IL-6, IL-10, and TNFα were determined in serum, and IL-17, IL-23, and IFNγ were determined in intestinal mucosa and serum. The FM groups did not differ in cell concentration, LA, or TP content (*p* > 0.05); FM-J28 had the highest EPS content. Spleen weight and colon length did not differ among the FM groups (*p* > 0.05). In the FM-J20 and PFM-J20 groups, IL-17 and IFNγ decreased, and the IL-10 concentration was enhanced (*p* < 0.05) at Week 6. IL-6, TNFα, IL-23, and IFNγ did not differ in serum and mucosa (*p* > 0.05), and IL-17 was lowest in FM-J28 and FM-J20. Therefore, FM appears to potentially play a role in decreasing the Th17 response. However, further studies are needed to elucidate the FM-mediated anti-inflammatory mechanisms in IBD.

## 1. Introduction

Inflammatory bowel diseases (IBDs) are characterized by chronic and uncontrolled inflammation in the intestinal mucosa. Different factors have been evidenced to affect the immune system at the mucosal level [1]. For example, inflammatory mediators such as cytokines play an important role in the adaptive immune response at the intestinal mucosal level. The modulation of biological cellular functions may initiate downstream signaling pathways and mediate cellular proliferation and differentiation [2]. In particular, Th1 and Th17 cells have been implicated in the development of IBD. Th1 is characterized by the presence of interferon-γ (IFNγ) and Th17 by the presence of interleukin (IL)-17, IL-21, and IL-22 [3]. Cytokines such as IL-6, TGF-β, and IL-23 promote the development of Th17 cells in IBD [4]. One study related Th1 and Th17 responses to the pathogenesis of IBD and suggested that Th1 cells may enhance the production of Th17 cells. In this previous study, a higher concentration of Th17 vs. Th1 cells was reported in a colitis model with CBirl TCR transgenic mice, which are immunodominant susceptible to flagellin microbiota [5]. In contrast, another study examining the cytokine profiles of Th1 and Th17 in a colitis model found that IL-4 and IL-10 levels were enhanced while IL-17 levels were reduced [6].

In another study, the pathogenic action of IL-23 was demonstrated. In IL-23R-deficient mice, reduced Reg3b protein expression in intestinal mucosa was shown to directly affect antimicrobial activity. In addition, IL-23-dependent Reg3b triggers an influx of IL-22, regulating the number of neutrophils in the lamina propia and restoring IL-22 secretion. This finding is important given the role of IL-23 in neutralizing the Th17 response in IBD [7].

Moreover, the administration of probiotics was shown to possibly modulate the inflammatory process through the Th1-Th2-Th17 response. Zheng et al. [8] reported that *Bifidobacterium breve* and *Lactobacillus rhamnosus* reduce Th17 and increase the Th2 cell subset in human peripheral blood mononuclear cells. In addition, the active components of probiotics were shown to be responsible for enhancing the numbers of CD4 + FoxP3 + regulatory T cells in mesenteric lymph nodes and for decreasing the cytokines tumor necrosis factor-α (TNFα), IFNγ, and IL-10 in Peyer’s patches and the large intestine. In another study, probiotics were shown to play an important role in the down-regulation of the nuclear factor kappa B (NFkB) pathway in RAW 264.7 cells to prevent TNFα expression in a lipopolysaccharide-induced model [9].

Several additional studies have documented the regulation of the immune response in IBDs by administering probiotics [10,11,12,13,14]. However, few studies have documented the effects of fermented milk on the Th17 response, which regulates various inflammatory processes at the intestinal level. In one case, Dahi-fermented milk containing a probiotic reduced myeloperoxidase (MPO) activity and TNFα, IL-6, and IFNγ levels [14]. Meanwhile, milk with fermented *Lactobacillus rhamnosus* GG reduced colonic inflammation and injury and stimulated the activation of epidermal growth factor receptor (EGFR) and protein kinase B (Akt), which may be attributed to the release of p40 and p75 proteins during fermentation [15]. 

These latter studies demonstrated the potential role of milk fermented with probiotics on the inflammatory process. However, the effect of fermented milk on the Th1/Th17 response has not been reported. The aim of the present study was to evaluate the effect of milk fermented with *Lactobacillus fermentum* (J20 and J28) on the Th1/Th17 response in a murine model of inflammation.

## 2. Materials and Methods

### 2.1. Preparation of Fermented Milk

The *Lactobacillus fermentum* strains J20 and J28 were cultured in MRS broth (De Man, Rogosa and Sharpe, Difco) for 12 h at 37 °C. Posteriorly, to elaborate the fermented milk, the strains were sub-cultured twice in commercial milk (1% *v*/*v*) and incubated for 24 h and 12 h at 37 °C. Then, commercial skimmed milk was inoculated (3% *v*/*v*) with the 12-h cultures and incubated for 48 h at 37 °C. Finally, the fermented milks (FMs) were placed in a cold water bath or submitted to heat treatment (75 °C, 15 min) to obtain pasteurized fermented milk (PFM), which was subsequently submersed in a cold water bath to inactivate bacteria. The samples were stored at 4 °C. A control pH treatment was prepared with acidified milk (AM) by adding 800 µL of lactic acid (~90%, Sigma-Aldrich, Mexico City, Mexico) to skimmed milk to obtain a similar lactic acid concentration as FM. 

### 2.2. Characterization of Fermented Milk

Bacterial cell concentration, lactic acid (LA) content, and total protein (TP) content were determined in the FMs and PFMs. The cell concentrations were determined at 48 h of fermentation by counts on plates of MRS agar. The LA and TP contents were determined by AOAC techniques 2000. The titratable acidity was expressed as percent LA titrated in 10 mL of fermented milk, using NaOH (0.1 N) and phenolphtalein as the indicator. TP was quantified by the Kjeldahl method using a nitrogen-to-protein conversion factor of 6.25.

### 2.3. Determination of Exopolysaccharide 

The exopolysaccharides (EPS) were precipitated from the supernatant of FM at 48 h of fermentation. Samples were centrifuged (3600× *g*, 60 min, 10 °C), and the supernatants were recovered. Afterwards, trichloroacetic acid solution (20% *v*/*v*, Sigma-Aldrich) was added to the supernatants, which were incubated for 2 h at 4 °C. Precipitated proteins were removed by centrifugation (3600× *g*, 60 min, and 10 °C). Next, the supernatants were treated with two volumes of cold ethanol, followed by 12 h of incubation at 4 °C. The EPS were recovered by centrifugation and posteriorly suspended in 1 mL of milli-Q water. The total EPS content was estimated for each sample by the phenol-sulfuric method using glucose as the standard [16]. 

### 2.4. Animal Study

Seventy mice C57Bl/6 (weight 30 g, six weeks old) were obtained from BIOINVERT (Mexico City, Mexico). The mice were randomly allocated into seven groups (*n* = 10/group) using a simple randomization procedure (computerized random numbers) based on their initial weight to obtain statistically equal groups (ANOVA, *p* > 0.05). Intestinal chronic inflammation was induced by the administration of dextran sulfate sodium (DSS). The mice were divided into the following treatment groups: (1) negative control (water); (2) DSS group; (3) AM + DSS (AM); (4) FM-J20 + DSS (FM-J20); (5) PFM-J20 + DSS (PFM-J20); (6) FM-J28 + DSS (FM-J28); and (7) PFM-J28 + DSS (PFM-J28). Half of the animals from each group were tested in a model of mild inflammation (six weeks) to evaluate the course of inflammation. The other mice were tested up to the end of the experimental period to evaluate chronic/systemic inflammation. Mice were housed in a controlled environment (22 °C, 12 h/12 h light/dark cycle) and fed a conventional diet and water *ad libitum*. This study, including the corresponding animal experiment, was approved by the Bioethics Committee of the Research Center for Food and Development (CIAD for its Spanish acronym), Hermosillo, Sonora, Mexico (CE/002/2015).

**Figure 1:**
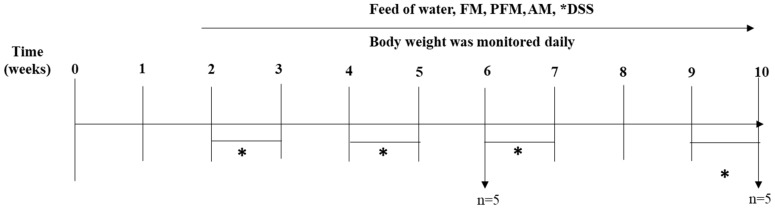
Experimental design. FM + DSS, PFM + DSS, and AM experimental groups of mice received daily treatments from Week 1 to Week 10. Mice received DSS in Week 2, 4, 6, and 9. *DSS administration period

Mice were intragastrically fed 800 µL/day/mouse of either FM or water (control groups) for 10 weeks. To induce chronic inflammation, mice were intragastrically fed with 3% (*w*/*v*) DSS (40 kDa; Sigma-Aldrich) dissolved in sterile water. Mice were treated with four cycles of DSS. For each cycle, mice were fed with 200 µL/day/mouse for seven days; subsequently, between each cycle, mice drank water normally for seven days [6] (Figure 1). Body weight and water and food consumption were recorded daily. Half of the animals per group were euthanized at Week 6, while the rest of the mice underwent two additional rounds of DSS administration and milk feeding and were euthanized at Week 10.

The body weight initial ranged from 27.2 ± 4.9 g to 30.0 ± 3.1 g. The body weight gain was 4.2 ± 9.2 g and was highest for water group and lowest for the LFJ28 group.

#### 2.4.1. Serum, Organ, and Mucosa Collection

After mice were euthanized (*n* = 5), the spleen and colon were removed. Spleens were weighed, and the colon lengths were measured. Blood samples were collected by cardiac puncture. The blood samples were allowed to clot at room temperature for 30 min before centrifugation (3500 rpm, 5 min, 4 °C), and the serum was collected and stored at −80 °C until further analysis. 

For the mucosal collection, segments of the distal colon were removed from mice, cut open longitudinally, and mixed with 1 mL of fresh RPMI 1640 medium (Sigma-Aldrich) supplemented with penicillin–streptomycin (1%). Then, the samples were incubated at 37 °C for 24 h. The culture supernatants were harvested by centrifugation (3600× *g*, 10 min, 10 °C), and stored at −20 °C until being assayed [17].

#### 2.4.2. Quantification of Cytokines 

Murine IFNγ minimum detectable dose (MDD 1.8 pg/mL), IL-17 (MDD, 5 pg/mL), IL-22, and IL-23 (MDD 2.28 pg/mL) were evaluated in samples of serum and mucosal tissues by ELISA (R&D System). Serum samples were also used for quantification of IL-6 (MDD 1.4 pg/mL), IL-10 (MDD 6.8 pg/mL), TNFα (MDD 0.9 pg/mL), IFNγ (MDD 0.5 pg/mL), and IL-17 (0.8 pg/mL) (CBA kits, R & D Systems, San Jose, CA, USA) by flow cytometry (BD FACSCantoTM II, San Jose, CA, USA).

#### 2.4.3. Histological Analysis

A histological examination of distal colon samples was performed by triplicate observations of three samples from the distal colon of each animal. Specifically, 0.5 cm of each sample were fixed in 10% buffered formalin, dehydrated in ethanol, paraffin embedded, and continuously sliced. Then, samples were deparaffinized, rehydrated, and stained with hematoxylin and eosin staining. The samples were evaluated under a 10× light-fluorescent microscope (LEICA DM2000, Leica Mycrosystems Inc., Chicago, IL, USA) to observe morphological changes to colonic tissue and the imagens were processed in the LEICA V2 program.

### 2.5. Statistical Analysis

EPS, LA, TP, cell concentration, spleen weight, and colon length were analyzed by one-way ANOVAs. Differences among means per group were analyzed using Tukey–Kramer tests and were considered significant when *p* < 0.05. The cytokine values were analyzed using the non-parametric Kruskal–Wallis test; the data were presented as medians and considered significant when *p* < 0.05. The cytokine concentrations were determined by ELISA using ELISAanalysis.com software; the concentrations from CBA kits were analyzed using the BD FACSArrayTM bioanalyzer (Becton Dickinson, San Jose, CA, USA) Statistical differences were analyzed in the Minitab V.16.1.1. package.

## 3. Results and Discussion

Several studies have reported that an anti-inflammatory response may be attributed to the presence of LA bacteria and their cell components [18]. Additionally, other components, such as EPS released during the fermentation process [19] and LA present in the matrix of FM [20], may generate an anti-inflammatory response. Therefore, cell concentration, LA, EPS, and TP content from both FMs were analyzed and are described in Table 1. Notably, several bacteria are known to synthesize EPS; the EPS are associated with the cell surface or are present as slime. Some studies have reported on the role of EPS in protecting against desiccation and toxic compounds. In addition, an important characteristic of probiotics is the presence of EPS, which allows adhesion to solid surfaces and biofilm formation [16]. 

Under fermentation conditions, it was previously suggested that EPS production was associated with bacterial growth. In the present study, EPS production was determined at 48 h of fermentation, which corresponded with the highest cell concentration (9 log CFU/mL) for both FMs. FM-J28 showed the highest EPS concentration (*p* < 0.05). Some studies have described other conditions that may influence EPS production, such as type of bacteria, carbon source, and temperature of incubation [21]. Moreover, it has since been suggested that EPS may enhance the anti-inflammatory effect [19]. We determined that *Lactobacillus fermentum* was able to produce EPS under these fermentation conditions even though cell concentration, LA, and TP did not show significant differences (*p* > 0.05) among the milks fermented with different strains or the milks fermented with different strains that underwent pasteurization. 

### 3.1. Anti-Inflammatory Response

**Figure 2:**
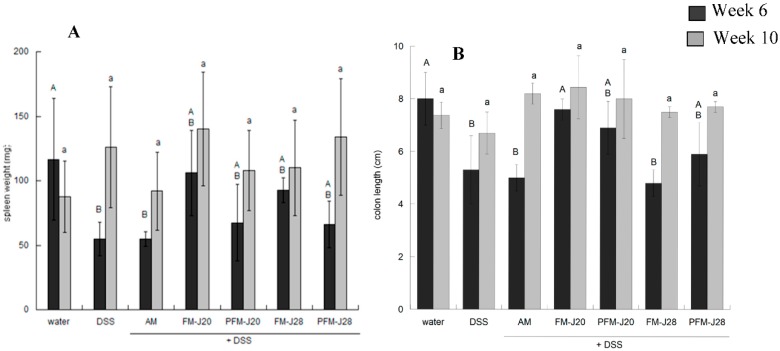
Effect of dextran sulfate sodium and fermented milk administration on spleen weight (**A**) and colon length (**B**) at Weeks 6 (black) and 10 (grey). Bars with an uppercase letter indicate statistical differences at Week 6 among treatments, and bars with a lowercase letter indicate statistical differences at Week 10 according to the Tukey–Kramer test (*p* < 0.05). The values are the means ± SD (*n* = 5).

Previous studies have demonstrated that, after four or five cycles of DSS administration, inflammation at the intestinal level may occur, affecting physiological parameters such as weight loss, intestine length, and the weight of other organs [22]. In the present study, the spleen of the DSS group had a significantly lower weight (*p* < 0.05) with respect to the water group at Week 6; however, it was not different (*p* > 0.05) compared to the groups administered with either FM. Conversely, in the end assay, the spleen weight did not significantly differ among groups (*p* > 0.05) (Figure 2). Furthermore, spleen weight at Weeks 6 and 10 for each group presented statistical differences (*p* < 0.05); the highest weight was found at Week 10 for the groups administered with DSS or FM + DSS.

The importance of the spleen as a secondary organ in the immune response may be highlighted. It is one of the sites where the immune response may be regulated and also stores immunological cells such as B and T cells, dendritic cells, and macrophages, which exert discrete functions [23]. Studies have demonstrated that, during inflammatory processes, spleen weight may increase because of immune activation or infection, leading to the hyperactivity of the spleen [12]. In the present study, the spleen weight increased in all treatments at Week 10, with exception of the water group; however, these differences were not significant (*p* > 0.05), indicating that the effect of DSS and FM treatments may be limited.

Colon length did not differ significantly among all groups (*p* > 0.05) nor between Weeks 6 and 10, which may indicate a low response to the inflammatory process during the study period.

The administration of DSS is known to reproducibly induce mild intestinal inflammation and the development of ulcers in mice, increasing neutrophil counts in the intestine [24]. In this regard, the capacity of Th17 cells to secrete IL-17 but not IFNγ or IL-4 has been described; these prior cells play an important role in the mediation of the inflammation process and tissue destruction [25,26]. Therefore, it is important to know their functions and develop strategies that block their response at local level, principally in IBDs [27].

**Figure 3:**
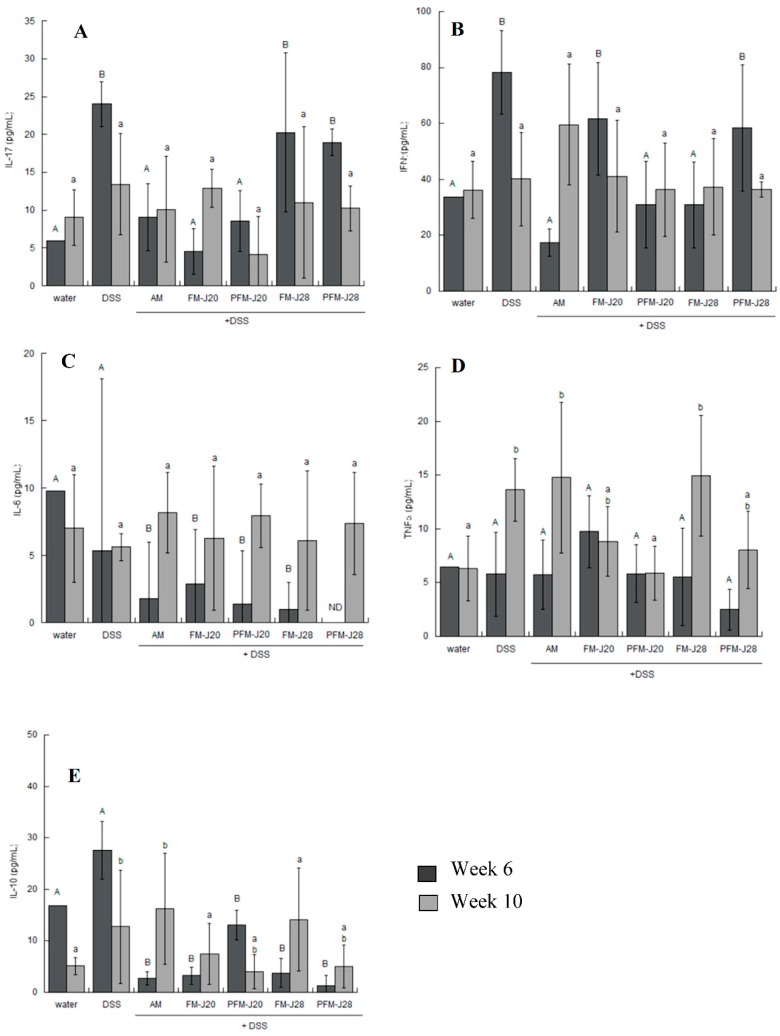
Effects of DSS and fermented milk administration on: IL-17 (**A**); and IFNγ (**B**) in serum samples, determined by ELISA; and on: IL-6 (**C**); TNFα (**D**); and IL-10 (**E**), determined by flow cytometry at Weeks 6 and 10. Bars with an uppercase letter indicate statistical differences at Week 6 among treatments, and bars with a lowercase letter indicate statistical differences at Week 10 (*p* < 0.05) according to the Kruskal–Wallis test. The values correspond to medians ± interquartile ranges for *n* = 5.

The serum cytokine profiles at Week 6 showed a significant increase in IL-17 in the groups subjected to DSS-induced inflammation with respect to the water group (*p* < 0.05) (Figure 3A). Meanwhile, the concentration of IFNγ in the DSS group was enhanced compared to the water group at Week 6, yet decreased by Week 10 (*p* < 0.05) (Figure 3B). The concentration of IFN-γ in serum samples was enhanced at Week 6 for the DSS groups FM-J20 and PFM-J28. These values corresponded with an enhancement in the Th1 response, as various reports have documented. The inflammation process, as based on IL-17, was sufficient at Week 6; hence, by Week 10, the organisms were possibly able to regulate the inflammation process. In our study, for example, the FM-J28 group at Week 6 showed enhanced IL-17 but not IFN. This is a possible response mechanism to a low concentration of IFNγ. Finally, IL-22 and IL-23 were not detected. 

The groups administered with FM-J20 and PFM-J20 showed the lowest concentration of IL-17 (*p* < 0.05) at Weeks 6 and 10. FM-J20 and PFM-J20 differed in the presence of viable bacterial cells, yet both FMs were able to decrease the concentration of IL-17, suggesting that different components from FM are involved in this effect. However, no statistical difference was encountered at Week 10 with respect to the DSS group (*p* > 0.05). Furthermore, IL-6 and TNFα did not show statistical differences among treatments and the control (DSS and water) at Weeks 6 and 10 (*p* > 0.05) (Figure 3C,D). However, the concentration of IL-10 cytokine did significantly differ (*p* < 0.05) (Figure 3E); the groups administered with PFM-J20 and FM J28 presented the highest concentration at Weeks 6 and 10, respectively. 

It was previously reported that the expression of the genes that encode for IL-6, IL-1β, IL-23A, TGFβ, and STAT3 are involved in the differentiation of Th17 cells in a DSS-induced inflammation model [28]. Furthermore, studies have demonstrated that Th17 cells require specific cytokines such as IL-23, which mediates the expansion of IL-17. On the other hand, low levels of IFNγ and IFNα may enhance the gene expression of IL-17 and IL-17-producing cells generated by IL-23 stimulation [29].

The findings reported in the present work are in agreement with those of other studies. For example, the administration of VSL#3 probiotics decreased TNFα and IL-6 and increased IL-10 serum levels in a DSS-induced colitis model of inflammation [10]. However, the concentration of inflammatory cytokines in our study is minor compared with other studies, which may indicate that the response following FM intake is lower in our inflammation model. Different compounds derived from FM are responsible for the anti-inflammatory effect [30,31]. For instance, milk fermented with *Lactobacillus rhamnosus* GG (LGG) was found to reduce colonic inflammation and injury and to also stimulate the activation of EGFR and Akt pathways while suppressing cytokine-induced apoptosis. This effect was attributed to the soluble proteins p40 and p75 present in milk fermented with LGG [15]. Furthermore, the immunoprotective effect of probiotic Dahi containing *Lactobacillus acidophilus* LaVK2 and *Bifidobacterium bifidum* BbVK3 on DSS-induced ulcerative colitis in mice was demonstrated to significantly reduce TNFα, IL-6, and IFNγ cytokines in colonic tissue [14].

Several reports have shown the different mechanisms of action involved in the anti-inflammatory effect of probiotics, but few studies have demonstrated possible pathways at the cellular level. One possible mechanism of action may be related with cell viability or cell wall components, which can be internalized into M cells, interact with dendritic cells, and then down-regulate the production of the pro-inflammatory cytokines IL-1β, IL-6, IL-17, TNFα, and IFNγ [32,33]. Furthermore, bioactive peptides have shown anti-inflammatory activity by down-regulating LPS-induced cytokine production in monocytes cells via the NF-kB pathway [34]. 

Moreover, LA may induce different inflammatory responses in LPS-stimulated RAW 264.7 cells. Some studies have reported its association with metabolic acidosis, which frequently complicates sepsis and septic shock and may be deleterious for cellular function. In this study, the AM group showed no statistical difference (*p* > 0.05) with respect to the control group and FM groups; however, it is important to carry out further studies on LA and its role in inflammatory processes. 

**Figure 4:**
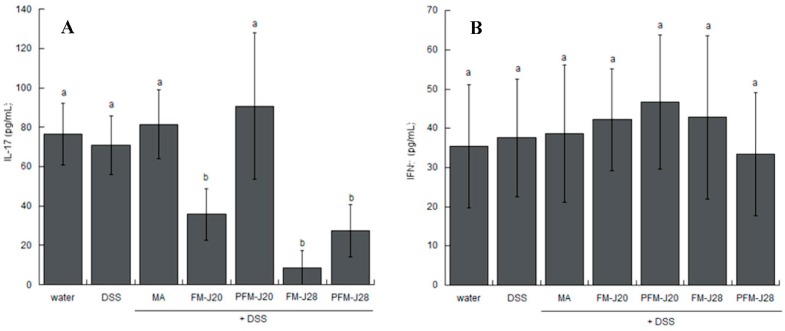
The effect of DSS and fermented milk administration on IL-17 (**A**) and IFNγ (**B**) determined by ELISA of colonic mucosa at Week 10. Different letters show statistical differences (*p* < 0.05) among treatments according to the Kruskal–Wallis test. The values are medians ± interquartile ranges (*n* = 5).

In the determination of cytokines in samples of intestinal mucosal (Figure 4), FM-J20, FM-J28, and PFM-J28 showed statistical differences (*p* < 0.05) among treatments with respect to the controls (DSS and water). However, IFNγ levels were not statistically different (*p* > 0.05), and IL-23 was not detected at the mucosal level. These results correspond with those reported in other studies wherein the presence of IFNγ inhibited IL-17 [5]. IL-23 is important for the expansion, stabilization, and conditioning of the Th17 response; hence, the IL-23/17 axis may be considered as a hallmark and an attractive probiotic therapeutic target in IBD [32]. The study by Jadhav et al. [14] showed the lowest concentrations of TNFα, IL-6, and IFNγ in samples of colonic tissue from mice administered with Dahi (fermented dairy) + DSS as well as a group administered probiotic + DSS.

This effect has also been observed in mice following the administration of milk fermented with *Bifidobacterium* strains. In particular, these mice showed a lower concentration of TNFα in culture supernatants, while IL-10 increased in the FM group and also reduced the histological score compared to treatments with saline and unfermented milk [35]. A possible mechanism for the anti-inflammatory effect may be related to the inhibition or modulation of the expression of several genes such as NF-kB, RELB, and TNFα, which have been reported as active during inflammation processes [36].

The binding of IL-10 to its receptor triggers phosphorylation-dependent activation of the transcription factor STAT3, which upregulates the gene expression of members of the SOCS family and proinflammatory cytokines such as TNFα and macrophage inflammatory protein 2. However, this process is possibly inhibited by the presence of bacteria or other components, such as EPS [37].

#### Histological Analysis

**Figure 5:**
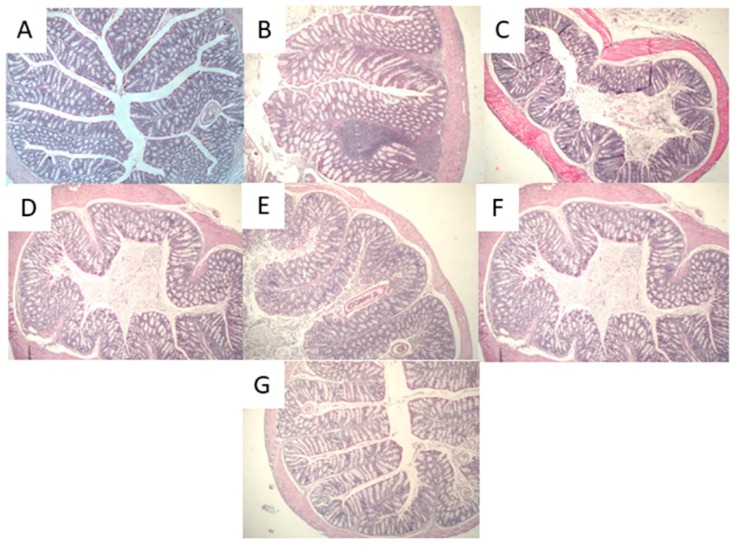
Histological analysis of cross sections of colon tissue samples stained with hematoxylin–eosin (10×): (**A**) water control group; (**B**) DSS group; (**C**) AM + DSS group; (**D**) FM-J20 + DSS group; (**E**) PFM-J20 + DSS group; (**F**) FM-J28 + DSS group; and (**G**) PFM-J28 + DSS group.

The DSS inflammation process is characterized by histological findings such as edema, infiltration of inflammatory cells into both the mucosa and submucosa, and destruction of epithelial cells [28]. Our results indicated that neither the administration of DSS or fermented milk affected the structure of mucosa or submucosa (Figure 5). A low number of inflammatory cells infiltrated (Figure 5B) but did not result in mucosal injury. Therefore, the histological analysis of samples confirmed that the inflammation process was not extensive; this finding may suggest that the presence of pro-inflammatory cytokines did not cause extensive changes. On the other hand, some studies have suggested that invasion of inflammatory cells into the mucosa produces increased concentrations of inflammatory cytokines such as TNFα, which then induce the expression of genes associated with inflammation [10]. In addition, DSS may induce the expression of COX-2, an enzyme responsible for the formation of prostanoids [38] that has been shown to be specifically induced in epithelial cells under IBD conditions [39].

These findings show that FM administration may prevent intestinal inflammation by stabilizing mucosal immunity through different components or metabolites derived during the fermentation process [13]. In particular, the present results show that the administration of FM could mediate the Th1/Th17 response, but more studies are required to determine the possible metabolites involved and the activation pathways. The interaction of derivative compounds from fermentation, such as LA and EPS, as well as the presence of bacteria could result in an anti-inflammatory response that stimulates IL-10 production and inhibits TNFα despite the interaction of TLR2 and TLR4 and activation of NF-kB. For example, in one previous study, the presence of LA and *Lactobacillus casei* Shirota culture supernatants suppressed phosphorylation and degradation of I-kB-α [40].

## 4. Conclusions

In the present study, several strategies to reduce inflammation in an IBD model employing milk fermented with *Lactobacillus* strains were evaluated. The administration of milk fermented with *Lactobacillus fermentum* possibly decreased the inflammatory response at Week 6 because of the metabolites or cell components present in this product. However, further studies are needed to determine the modulation of Th1/Th17 by fermented milk. The findings in the present study show the potential regulatory effect of FM on the inflammatory process, although this effect was minor, possibly as a result of irregularity in the inflammatory process. Future studies are needed to establish an adequate model of inflammation that allows the Th17 response to be evaluated considering the other biomarkers involved in this response, including chemokines, transcription factors, and metabolites released during fermentation, as well as cell differentiation, which may all be responsible for promoting an anti-inflammatory response.

## Figures and Tables

**Table 1:** Cell concentrations, total protein in fermented milk and metabolites derived from the fermentation process.

Fermented Milk	Cell Concentration (Log CFU/mL)	Lactic Acid (%)	Exopolysaccharides (mg/mL)	Total Protein (%)
FM-J20	9.0 ± 0.01 ^a^	0.72 ± 0.01 ^a^	50.99 ± 0.10 ^a^	2.80 ± 0.04 ^a^
PFM-J20	ND	0.75 ± 0.03 ^a^	61.00 ± 0.15 ^a^	2.71 ± 0.09 ^a^
FM-J28	9.04 ± 0.01 ^a^	0.90 ± 0.01 ^a^	79.59 ± 0.05 ^b^	3.01 ± 0.05 ^a^
PFM-J28	ND	0.87±0.01 ^a^	77.30 ± 0.01 ^b^	2.88 ± 0.07 ^a^

ND = Not detected. The values are the means ± SD (*n* = 3). Different letters for a same parameter indicate statistical differences (*p* < 0.05) among dietary groups. Statistical differences were established by one-way ANOVAs and Tukey–Kramer Post Hoc tests. Raw (FM) and pasteurized (PFM) milks were fermented with *Lactobacillus fermentum* strains J20 and J28.
